# RASSF-1A modulates proliferation-mediated oral squamous cell carcinoma progression

**DOI:** 10.1186/s12935-019-0932-9

**Published:** 2019-08-13

**Authors:** Jianli Sun

**Affiliations:** Department of Oral and Maxillofacial Surgery, Henan Provincial Hospital of Stomatology, The First Affiliated Hospital of Zhengzhou University, No. 1 Eastern Jianshe Road, Zhengzhou, 450000 Henan China

**Keywords:** RASSF-1A, Oral squamous cell carcinoma, CyclinD1, SCC9

## Abstract

**Background:**

To investigate the expression of RASSF-1A in oral squamous cell carcinoma (OSCC) and adjacent tissues, and to explore its mechanism of action in the development of OSCC.

**Methods:**

RASSF-1A and proliferation-related protein expression in clinical and OSCC mouse models were detected by qPCR and western blot. In vitro experiments were used siRNA knockdown of RASSF-1A gene in SCC9 cells to detect cell proliferation, migration and apoptosis. In vivo experiments were performed using adenovirus overexpressing RASSF-1A gene in mice and observing tumor growth.

**Results:**

The results of qPCR and western blot showed that the expression of RASSF-1A gene was decreased in OSCC, and the expression of CyclinD1 protein was increased. The results of co-immunoprecipitation showed that the two proteins were significantly combined in the oral cancer cell line. Knocking down the RASSF-1A gene in SCC9 cells promotes cell migration and proliferation, while reducing apoptosis and increasing CyclinD1 protein expression. Overexpression of RASSF-1A gene in mice reduces tumor volume and inhibits CyclinD1 protein expression.

**Conclusions:**

Low expression of RASSF-1A gene in OSCC promotes the expression of CyclinD1 protein and tumor growth.

## Background

Oral cancer is one of the ten most common malignant tumors in the world, accounting for 5% of systemic malignancies, 90% of which are epithelial-derived squamous cell carcinoma [1]. In recent years, the incidence of oral squamous cell carcinoma (OSCC) is increasing and the age of onset is getting younger [2]. Quamous cell carcinoma, abbreviated as squamous cell carcinoma, also known as epidermal carcinoma, is a malignant tumor that occurs in the epidermis or accessory cells. The cancer cells have different degrees of keratinization, and are more common in areas covered with squamous epithelium, such as skin, mouth, lips, esophagus, cervix, vagina, etc. [3]. OSCC is a common malignant tumor of the head and neck. The World Health Organization predicts that the incidence of OSCC will continue to rise in the next decade, and OSCC has become a disease with high morbidity and mortality. The world public health problem encourages people to further study the factors that influence the prognosis of the disease [4]. Despite significant advances in cancer research over the past few decades, OSCC is still a worldwide malignancy. Usually, cancer begins with a single cell mutation in a somatic cell that leads to further proliferation, which activates the protooncogene and becomes an oncogene [5]. Immunohistochemistry has been used to detect potential markers of head and neck tumors, which contribute to the diagnosis and prognosis of the disease. Epigenetic modification refers to a change in the expression and function of a gene without a change in the DNA sequence and a heritable phenotype. It plays an important role in gene expression, regulation, and inheritance, and plays an important role in the process of tumorigenesis. The regulatory mechanisms of epigenetic modification are methylation of DNA, methylation and acetylation of histones, and regulation of non-coding RNA [6]. Epigenetic modification can lead to silencing or activation of genes. If epigenetic modification abnormalities in somatic cells lead to abnormal expression of certain genes, such as oncogene activation and tumor suppressor gene inactivation, abnormal proliferation of somatic cells. Recent studies have also shown that the occurrence of many malignant tumors is closely related to the epigenetic disorder of the cellular genome. This also provides new ideas for the study of molecular markers and therapeutic targets for malignant tumors at the epigenetic level [7]. The diagnosis of previous oral cancer is mainly based on clinical manifestations, imaging, tumor marker levels or biopsy, and tumors have formed at the time of diagnosis [8]. However, normal cells have developed signs of malignant transformation before the formation of tumors. If the epigenetic test is performed on the tissues, prediction or diagnosis can be made at an early stage or even before the cancer, and early prevention or treatment can improve the survival rate and improve the prognosis [9]. One study generated stable head and neck squamous cell carcinoma (HNSCC) cell lines ectopically expressing the c-Fosgene. Exogenous expression of c-Fos in nontumorigenic MDA1386Tu cells makes these cells tumorigenic in nude mice. Furthermore, subcutaneous transplantation of c-Fos-overexpressing Cal27 cells (tumorigenic) into immunocompromised mice enhanced tumor growth as compared with parental cells. Mechanistic investigations demonstrated that c-Fos overexpression enhanced the epithelial–mesenchymal transition state and expression of CSC markers (Nanog, c-Myc, Sox2, and Notch1). Ectopic expression of c-Fos in HNSCC cells also displays increased sphere formation. We further observed that overexpression of c-Fos increased the expression of pERK and cyclin D1 in HNSCC cells [10].

Since its discovery, the RASSF-1A gene has been extensively studied. A number of studies have shown that RASSF-1A is expressed almost in normal tissues and organs, but there are expression defects in various solid tumors [11]. OSCC is a multi-factor participation, a common malignant tumor with multiple genes, and the inactivation and loss of tumor suppressor genes are closely related to its occurrence and development [12]. The study found that the heterozygous loss of alleles often occurs in the short arm of chromosome 3 in OSCC. It is speculated that there may be tumor suppressor genes related to OSCC in chromosome 3p21. RASSF1 was reported from chromosome 3p21.3 locus novel candidate tumor suppressor gene [13]. It has a variety of different mRNA splicing bodies, RASSF1A is one of the main ones, which is positive 100% expression in normal tissues, but often expressed in lung cancer, breast cancer, nasopharyngeal cancer, kidney cancer, prostate cancer and bladder cancer. The main mechanism of aberrant methylation of the RASSF1A gene in the 5′ CpG island of promoter has been found in a variety of tumors, but there are few reports about RASSF1A gene methylation status and transcriptional level expression in oral precancerous lesions and squamous cell carcinoma [14]. This study was designed to investigate the expression of RASSF-1A in OSCC and adjacent tissues, and to investigate the mechanism of RASSF-1A gene in OSCC carcinogenesis, which looks for new directions for the treatment of OSCC.

## Methods

### Clinical patient specimens and animal model establishment

A total of 6 specimens of clinical oral squamous cell carcinoma (OSCC group) were collected, including paracancerous tissues (metastatic OSCC group), and 6 patients in the normal group (oral benign proliferative tissue) were collected. All patient specimen collection methods were reviewed and approved by the Ethics Review Committee of Henan Provincial Hospital of Stomatology, the First Affiliated Hospital of Zhengzhou University.

A total of 12 female C57BL/6 SPF grade mice (provided by Shanghai Experimental Animal Center, Chinese Academy of Sciences) weighing (20 ± 5) g, age of 50 days, were selected in this research. They were kept at room temperature 18 to 25 °C, humidity 30 to 50% with standard feeding under 24 h light and dark cycle conditions. The mice in the model group were prepared with reagent 4NQO (Sigma, USA), and was prepared at a concentration of 2% with 1,2-propanediol and stored in a refrigerator at 4 °C at dark. Oral cancer model mice were fed with 200 mg/L 4-NQO water for 14 weeks. After stopping the drug, they were switched to normal tap water for 45 weeks. Ordinary tap water was sterilized to make a concentration of 100 μg/mL in a dark bottle [15]. The mouse was injected with adenovirus overexpressing RASSF-1A gene in mice before the establishment of the cancer model (RASSF1A-V group, n = 6), and the control mice were injected with no-load virus (vector group, n = 6). The study was approved by the Ethics Committee of Henan Provincial Hospital of Stomatology, the First Affiliated Hospital of Zhengzhou University.

### Cell culture

Human tongue squamous cell carcinoma cell line SCC9 was purchased from ATCC, USA and cells were cultured in Dulbecco’s Phosphate Buffered Saline medium (DMEM) containing 10% fetal bovine serum (FBS; Gibco, USA). The cells were cultured at 37 °C in 5% CO_2_ atmosphere, and the growth status of cells was evaluated under a microscope. Cells were grown to 85 to 90% confluence, cells passaged, and subjected to trypsin digestion. SiRNA was used to knock down the RASF-1A gene in cells (RASSF1A-siRNA group) and control the addition of empty siRNA (non-siRNA group) into cells, and siRNA transfection efficiency was 30–40%. The blank control group was SCC9 cells without any treatment (SCC9 group). Knockdown was carried out for 72–96 h as specified.

### Apoptosis

SCC9 cells were transfected with control and RASSF-1A siRNA for 72 h. Cell pellets were collected with 0.05% trypsin–EDTA (Gibco), neutralized with 2% FBS in PBS, and washed with cold PBS. Apoptosis was assessed using the FITC Annexin V Apoptosis Detection Kit I (BD Pharmingen). Briefly, cells were resuspended in 1X Annexin Binding Buffer at 1 × 10^6^ cells/mL, stained with PI and FITC Annexin V, collected on a LSRII flow cytometer (BD Biosciences), and analyzed with FCS Express 6 software.

### Flow cytometric assays for cell cycle distribution

The cell cycle assay was performed by fixing the cells in 75% ethanol at 4 °C overnight and treated with 0.2% Triton X-100 and RNase before being stained with propidium iodide (PI) for 30 min in darkness. The cells were analyzed using an Accuri C6 flow cytometer (Accuri Cytometers Inc.; Ann Arbor, MI, USA), and the percentages of cells at each cell cycle phase were determined.

### Cell migration assay

SCC9 cells were cultured for 24 h, followed by digesting, counting, and suspension in serum-free 1640 medium. One hundred-microliter cell suspensions (2 × 10^5^ cells) were seeded in the upper chamber of a transwell unit with an 8.0 mm polycarbonate membrane (Millipore, Boston, USA) inserted in a 24-well plate, and 600 μL culture medium with 10% FBS was placed in the lower chamber. After the cells were incubated for 36 h at 37 °C, the cells on the top surface of the transwell chamber were removed with a cotton swab. The cells adhering to the lower surface were fixed with 4% paraformaldehyde for 30 min, stained with hematoxylin, and counted under a microscope in five randomly chosen fields.

### Wound-healing assay

SCC9 cells were seeded in 6-well plates at a density of 1 × 10^6^ cells per well. After the cells reached approximately 80–90% confluence, the cells were scratched with a 20 μL pipette tip for an additional 36 h. Images of the wound were recorded using a fluorescence microscope at two timepoints: immediately after wounding (0 h) and after culturing (36 h). The cells of both sides around the damaged area migrated toward the cell-free area. The wound widths of three different wound surfaces in each group were noted and subsequently measured using image J analysis software. The experiment was repeated three times.

### Western blot and co-immunoprecipitation assay

The total proteins from mice tumor tissues and SCC9 cells were isolated, and the protein concentrations were determined through a bicinchoninic acid assay. The proteins from whole-cell lysates were used for western blot using standard techniques. Anti-RASSF-1A (1:1000), anti-caspase3 (1:500), anti-CyclinD1 (1:500), anti-CyclinD2 (1:500), anti-Matrix Metalloproteinase-2 (MMP2) (1:1000), anti-GAPDH (1:1000) and anti-beta-actin (1:1000) antibodies (Cell Signaling Technology, Danvers, MA, USA) were used. The co-IP assay was performed using Protein A/G agarose (Beyotime, China), following the manufacturer’s instructions and normal rabbit anti-IgG (Abcam, UK) was used as a control antibody. Harvested samples containing input (protein pretreated with nothing), IgG (protein pre-treated with A/G agarose and rabbit anti-IgG), anti-RASSF-1A (protein pre-treated with A/G agarose and rabbit anti-RASSF-1A), and anti-CyclinD1 (protein pre-treated with A/G agarose and rabbit anti-CyclinD1) were analyzed by western blot.

### Immunohistochemistry

Sections were de-paraffinized and rehydrated. Heat-mediated antigen retrieval was performed in 0.01 M Citrate pH 6 at 95 °C. For human tissues, mouse anti-human RASSF-1A (CST, USA) was diluted 1:300 in antibody diluent (Thermo Scientific) and control rabbit IgG were used for immunoprecipitation and incubated overnight at 4 °C. Immunoreactivity was detected using the Vectastain Elite ABC and DAB peroxidase substrate kits.

### Real-time RT-PCR

The preparation of total RNA was obtained as described above, and cDNA synthesis was performed with a PrimeScript RT Reagent Kit (Takara, Otsu, Japan). The reaction was performed using Real MasterMix (SYBR Green; Tiangen, Beijing, China) in a total volume of 20 μL, containing 2 μL of cDNA, 9 μL of SYBR solution, 0.5 μL each of sense and antisense primers (10 μM), and up to 8 μL of ddH2O. The PCR amplification conditions for RASSF-1A were 95 °C for 3 min and 40 cycles of 95 °C for 10 s and 58 °C for 30 s. The amplification conditions for PCNA/CyclinD1/p38 MAPK were 95 °C for 3 min, then 40 cycles of 95 °C for 10 s, and 61.4 °C for 30 s. The primer sequences are shown in Table 1.

**Table 1:** Primer sequences

Gene	Primer sequences
RASSF1A	5′-TGGAGCGGGACACGAACG-3′
	5′-TGCGGCCTGACACTT-3′
PCNA	5′-ATGTTCGAGGCGCGCCTGGTC-3′
	5′-CTAAGATCCTTCTTCATCCTC-3′
CyclinD1	5′-ATGTTCGAGGCGCGCCTGGTC-3′
	5′-CTAAGATCCTTCTTCATCCTC-3′
p38 MAKP	5′-AACCCCAGAGCGAAATAC-3′
	5′-AAGAATGCCTCCTCACAC-3′

### Statistical analysis

GraphPad Prism 5.0 software was used for statistical analysis. Data were expressed as the mean ± standard deviation. Comparison betweentwo groups was performed using two-tailed t-test, unless otherwise indicated. Comparison between multi-groups was performed using one-way ANOVA with appropriate post hoc testing. There is a significant difference at p < 0.05.

## Results

### Low expression of RASSF-1A in tumor tissues of patients with oral squamous cell carcinoma

**Fig. 1:**
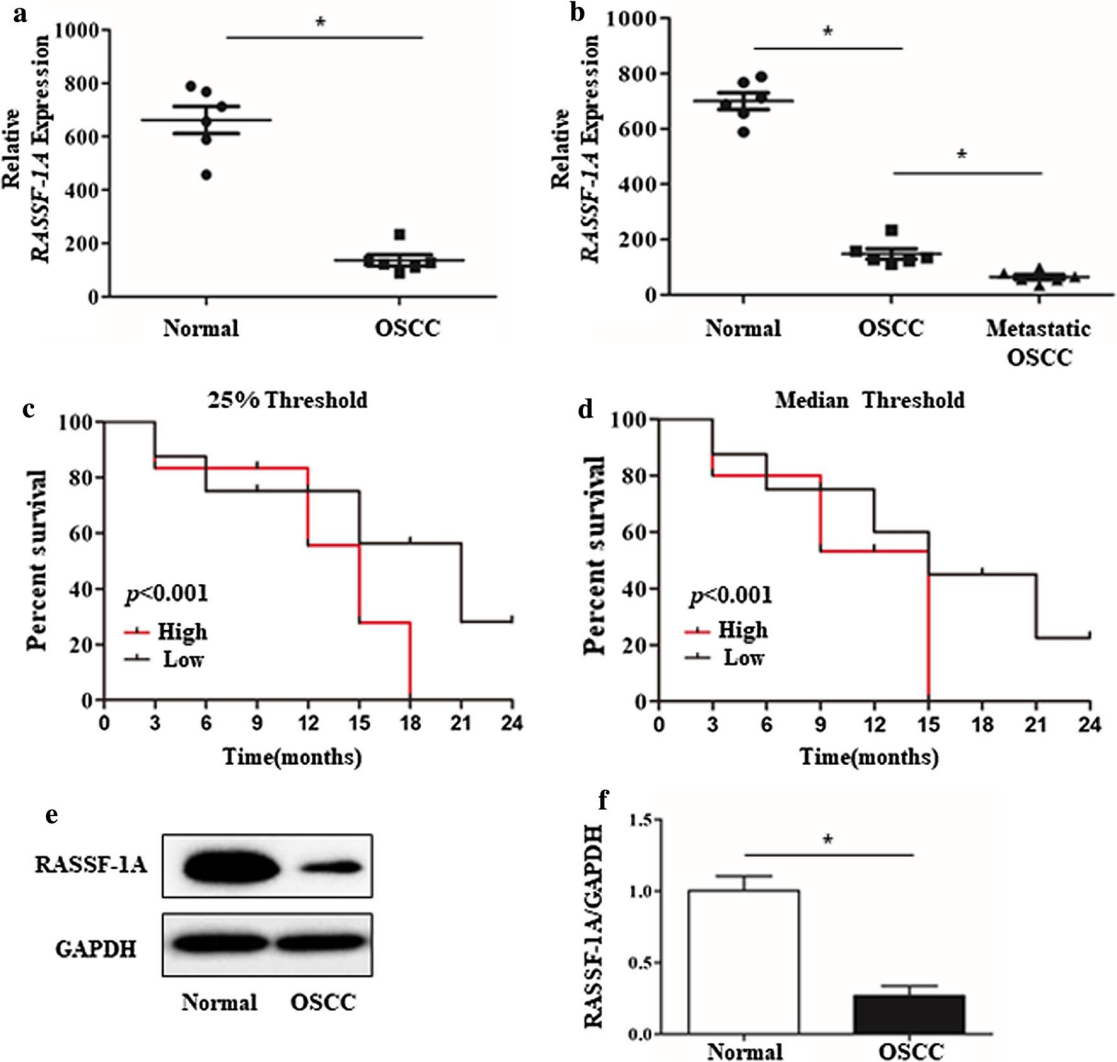
RASSF-1A gene is down-regulated in tumor tissues of patients with oral squamous cell carcinoma and is associated with patient survival. **a**, **b** Detection of RASSF-1A mRNA expression in tumor tissues and paracancerous tissues of patients with oral cancer by qPCR (n = 6 in normal group, n = 7 in OSCC group; *p < 0.05). **c**, **d** Kaplan–Meier plots for patients expressing RASSF-1A above (high) and below (low) the first quartile (left panel) and median (right panel) were constructed with Taylor et al. data via the web interface Betastasis. **e** Western blot analysis of RASSF-1A protein expression in tumor tissues of patients with oral cancer. **f** Statistical analysis of RASSF-1A protein expression in tumor tissues of patients with oral cancer by western blot (n = 6 in normal group, n = 7 in OSCC group; *p < 0.05)

In order to observe the expression of RASSF-1A gene in OSCC, we collected 6 tumors and adjacent tissues from patients with clinical OSCC and extracted tissue RNA and whole protein. Control group selected tongue tissue of patients with non-oral cancer. qPCR results showed that the expression level of RASSF-1A mRNA in oral cancer tissues and adjacent tissues was significantly lower than that in control tissues, with a significant difference (Fig. 1a, b). At the same time, the results of western blot showed that the expression level of RASSF-1A protein in oral cancer tissues was significantly lower than that in the control group, with a significant difference (Fig. 1e, f). Therefore, using data sets from clinical patient parameters, we found that low RASSF-1A mRNA expression was significantly associated with recurrence-free survival using the 50% and 25% cut-off thresholds (Fig. 1c, d).

### Knockdown of RASSF-1A promotes proliferation and migration of SCC9 cells

**Fig. 2:**
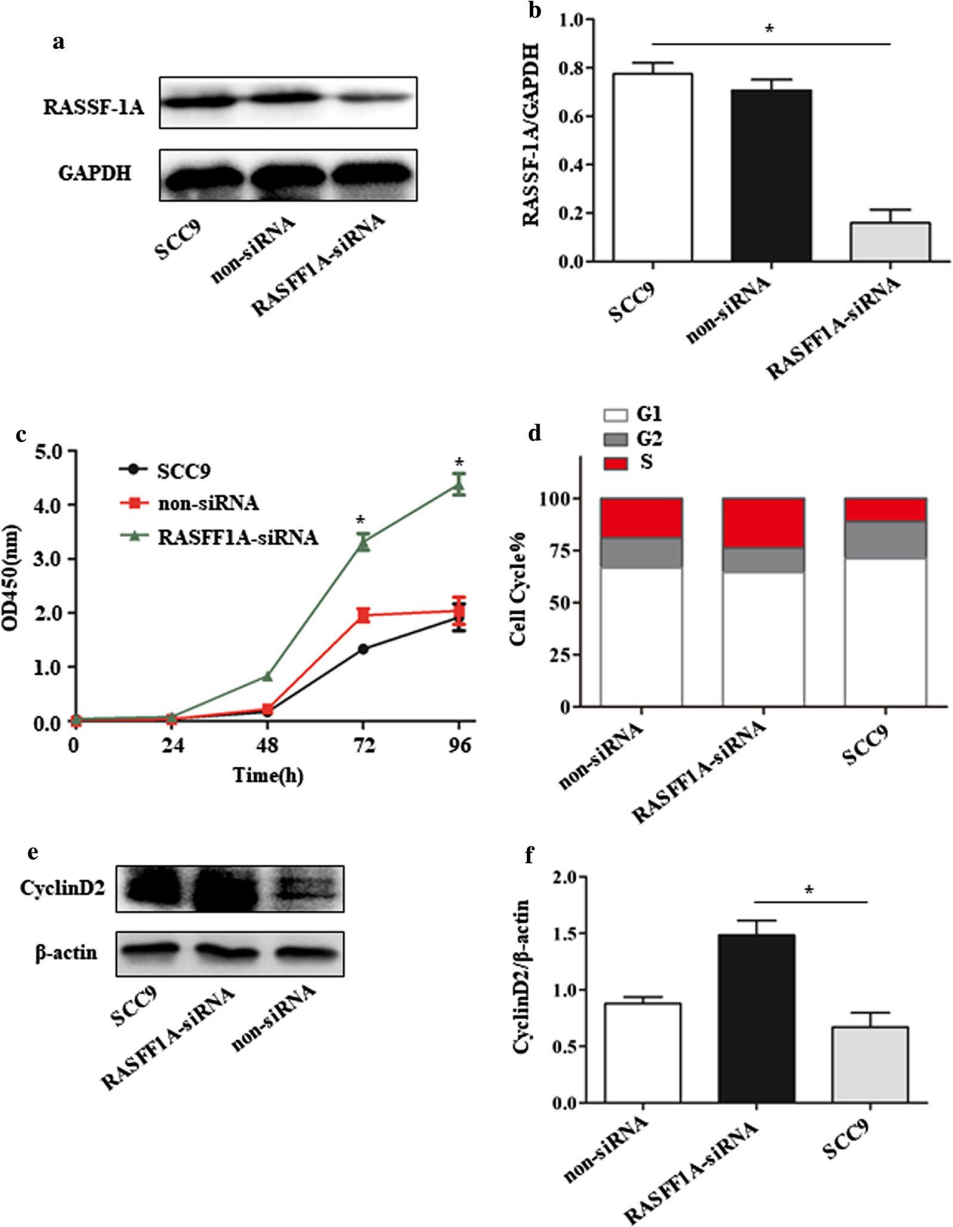
Knockdown of RASSF-1A gene in SCC9 cells promotes cell growth. **a** Western blot analysis of RASSF-1A protein expression in cells after siRNA interference. (SCC9: blank control group; non-siRNA: siRNA empty control group; RASSF-1A-siRNA: siRNA interference knockdown group). **b** Western blot results statistics. **c** CCK8 detects cell growth curve (at 72 h and 96 h *p < 0.05). **d** Flow cytometry to detect cell cycle distribution. **e** Western blot analysis of CyclinD2 protein expression in cells. **f** Western blot analysis of CyclinD2 protein expression (n = 6 in each group; *p < 0.05)

**Fig. 3:**
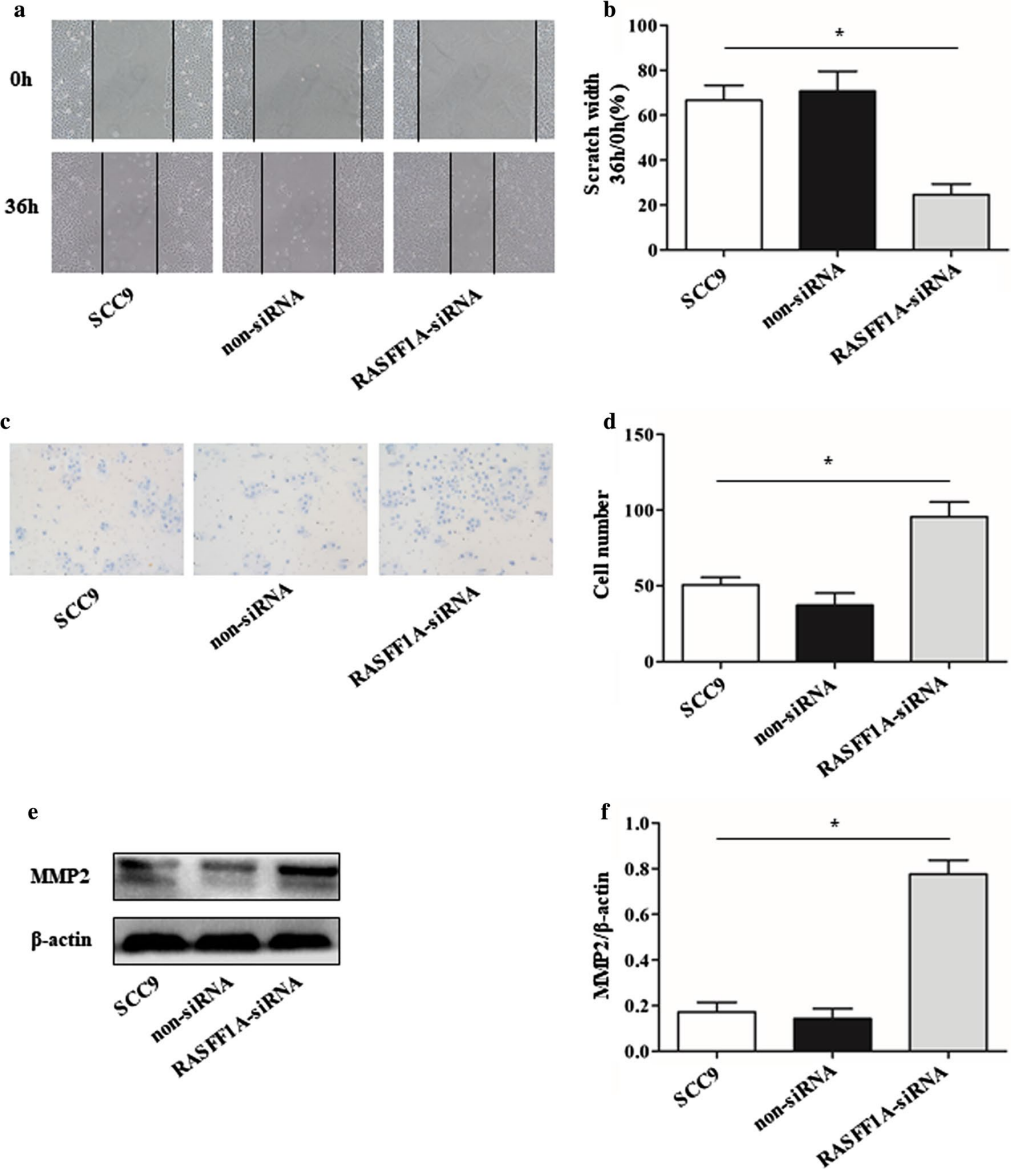
Increased cell migration ability after knockdown of RASSF-1A gene in SCC9 cells. **a** Scratch test to detect cell migration ability. **b** The width of the scratch between the cells of the different groups was counted at 0 and 36 h, and the width of the scratch was divided by the width of 36 h by the width of the scratch for 0 h (n = 6 in each group; *p < 0.05). **c** Transwell chamber for cell migration. **d** Crystal Violet staining counts the number of cells migrating through the transwell chamber after 36 h of cell culture (n = 6 in each group; *p < 0.05). **e** Western blot analysis of MMP2 protein expression in cells. **f** Western blot analysis of MMP2 protein expression (n = 6 in each group; *p < 0.05)

The RASSF-1A gene in SCC9 cells was knocked down by siRNA, and the control was performed using a blank control and empty siRNA. After siRNA interference for 72 h, the whole protein was extracted, and the expression of RASSF-1A protein was detected by western blot. The results showed that the expression of knockdown protein was decreased, with a significant difference (Fig. 2a, b). The results of CCK8 showed that the growth curve of SCC9 cells knocking down RASSF-1A gene was significantly higher than that of the control group at 72 and 96 h, with a significant difference (Fig. 2c). Flow cytometry showed that the percentage of cells knocking down the RASSF-1A gene in the S phase was significantly higher than that in the control group, indicating that the proliferation of SCC9 cells was significantly increased after knocking down the gene (Fig. 2d). Since the cell cycle regulatory protein is mainly composed of the Cyclin protein family, we examined the expression of CyclinD2 protein. It was found that knocking down RASSF1A protein increased the expression of CyclinD2 protein in cells (Fig. 2e, f). At the same time, we used cell scratch test and tanswell chamber test to detect changes in cell migration ability. The results of the scratch test showed that the percentage of the scratch width between the cells knocked down by the RASSF-1A gene at 36 h was significantly lower than that of the control group at 0 h, with a significant difference (Fig. 3a, b). After 36 h of cell culture in transwell chamber, the number of cell migrations with knockdown of RASSF-1A gene was significantly higher than that of the control group, with a significant difference (Fig. 3c, d). Matrix metalloproteinases (MMPs) affect the proliferation and migration of tumor cells. MMP2 is the major affector protein, so we examined the expression of MMP2 protein in cells. The results showed that the expression of MMP2 protein in cells was significantly increased compared with the control group after knockdown of RASSF1A gene (Fig. 3e, f).

### Knockdown of RASSF-1A gene can reduce apoptosis of SCC9 cells

**Fig. 4:**
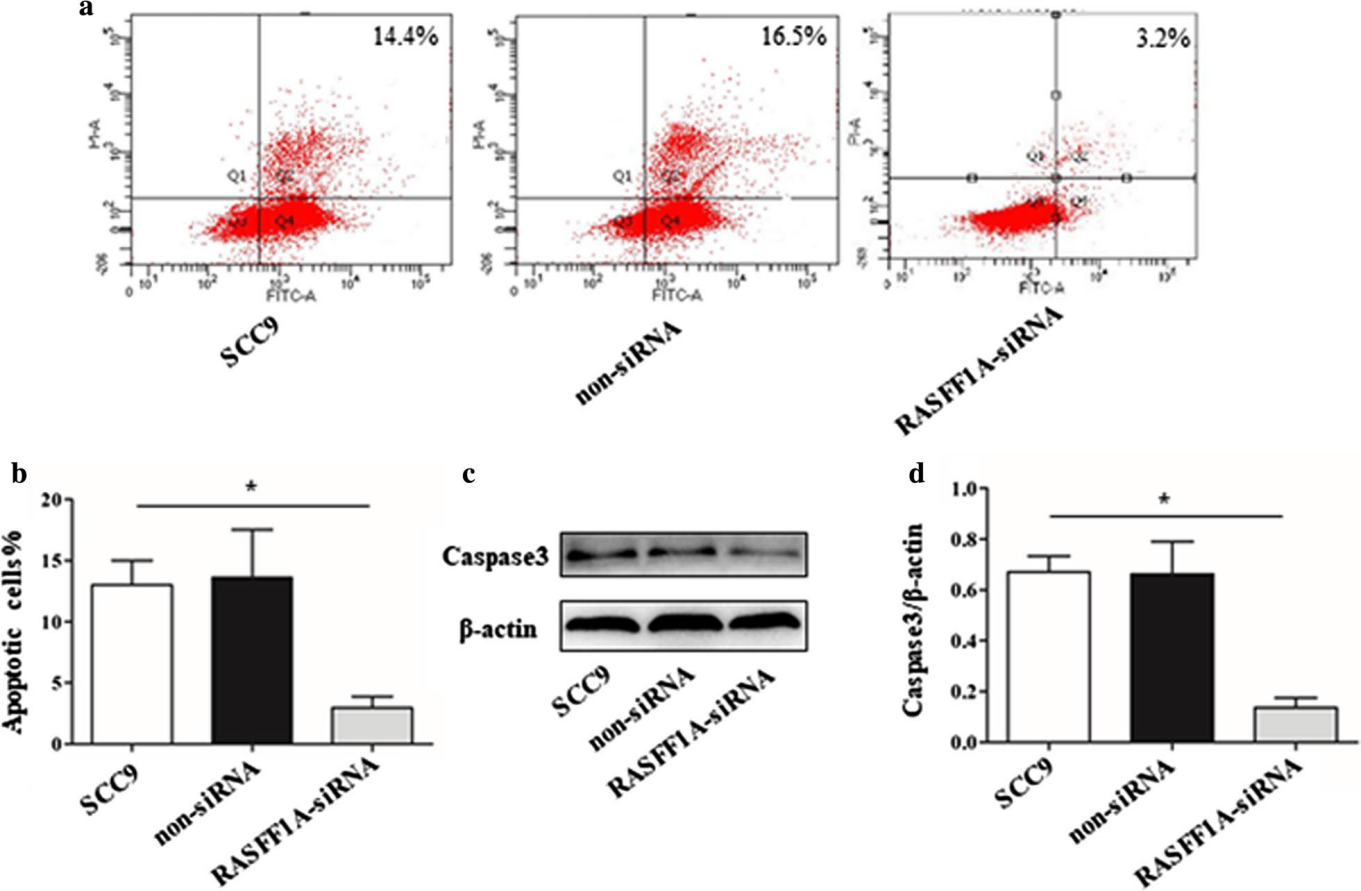
Decreased apoptosis after knockdown of RASFF-1A gene in SCC9 cells. **a** Flow cytometry to detect the percentage of positive expression of AV protein in cells. **b** Percentage of AV positive cells in statistical flow assay results (n = 6 in each group; *p < 0.05). **c** Western blot analysis of caspase3 protein expression in SCC9 cells. **d** Statistical analysis of the expression of caspase3 protein in cells by western blot (n = 6 in each group; *p < 0.05)

Usually cell proliferation and apoptosis are present at the same time, so we also detected SCC9 cells after knocking down the RASSF-1A gene. The positive expression rate of Annexin-V protein in flow cytometry showed that the positive expression rate of RASSF-1A gene was decreased, which was statistically significant compared with the control group (Fig. 4a, b). Caspase3 protein is a classic protein produced by apoptosis. Therefore, we used western blot to detect the expression of caspase3 protein in cells. The results showed that the expression of caspase3 protein in the knockdown group was significantly lower than that in the control group, with a significant difference (Fig. 4c, d).

### RASSF-1A regulates and binds the proliferating protein CyclinD1

**Fig. 5:**
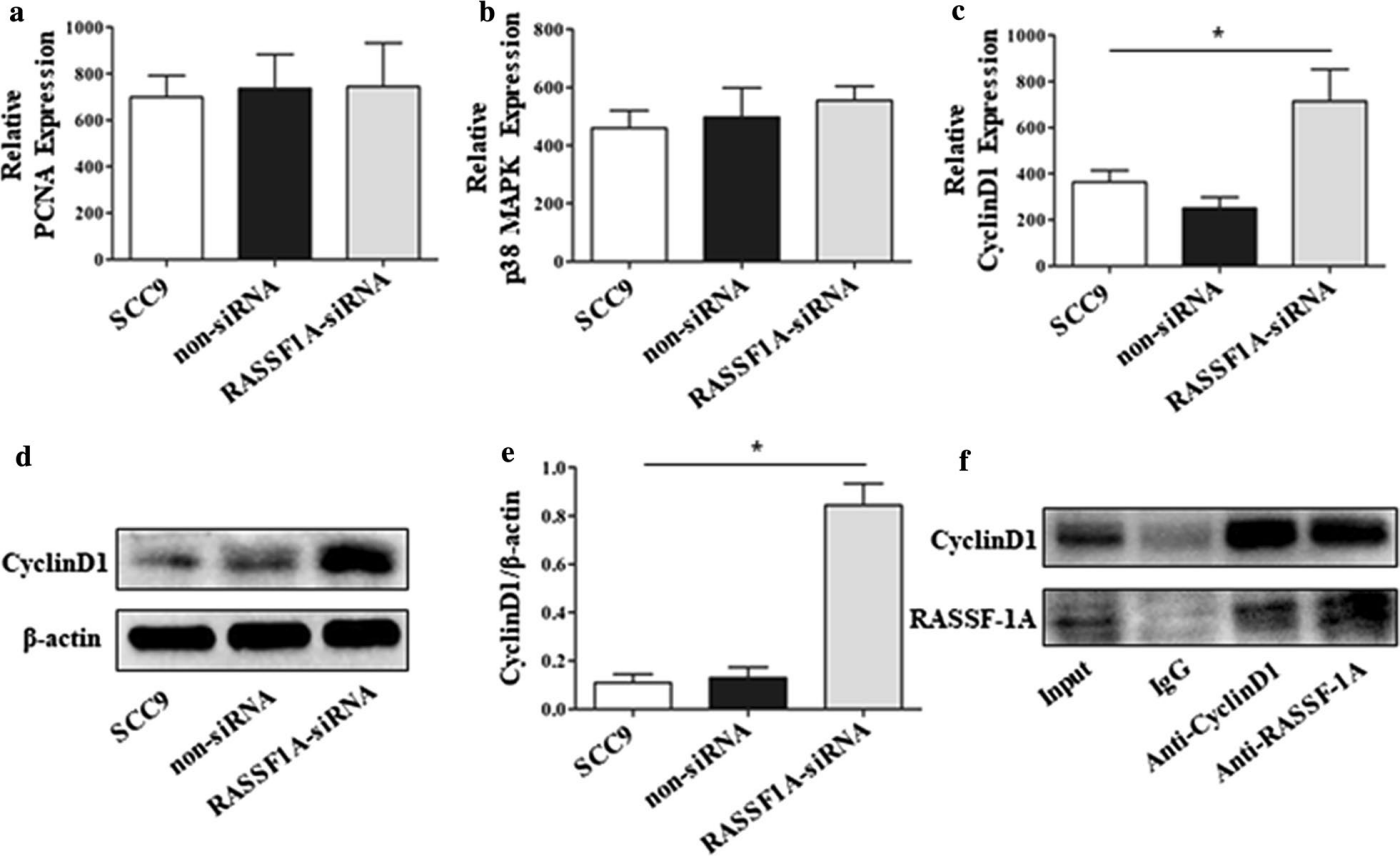
Knockdown of RASSF-1A gene in SCC9 cells promotes CyclinD1 protein expression. **a**–**c** qPCR detection of PCNA, p38 MAPK, CyclinD1 mRNA expression in cells. **d** Western blot analysis of CyclinD1 protein expression in cells. **e** Western blot analysis of CyclinD1 protein expression in cells (n = 6 in each group; *p < 0.05). **f** Co-immunoprecipitation assay for RASSF-1A protein and CyclinD1 protein binding in cells

From the above results, we can easily find that RASSF-1A gene plays an important role in regulating the growth and proliferation of OSCC, because we further tested the expression of several common downstream proteins related to tumor proliferation. The results of qPCR showed that CyclinD1 gene expression was the most obvious. After knocking down RASSF-1A gene on SCC9 cells, CyclinD1 gene and protein levels were significantly increased, and the difference was statistically significant compared with the control group (Fig. 5c, d). However, there was no significant difference in PCNA and p38 MAPK gene expression (Fig. 5a, b). The results of co-immunoprecipitation showed that RASSF-1A protein and CyclinD1 protein were significantly combined in SCC9 cells, indicating that RASSF-1A may have a certain regulatory effect on CyclinD1 gene (Fig. 5e).

### Overexpression of RASSF-1A gene in mice reduces tumor growth and inhibits CyclinD1 protein expression

**Fig. 6:**
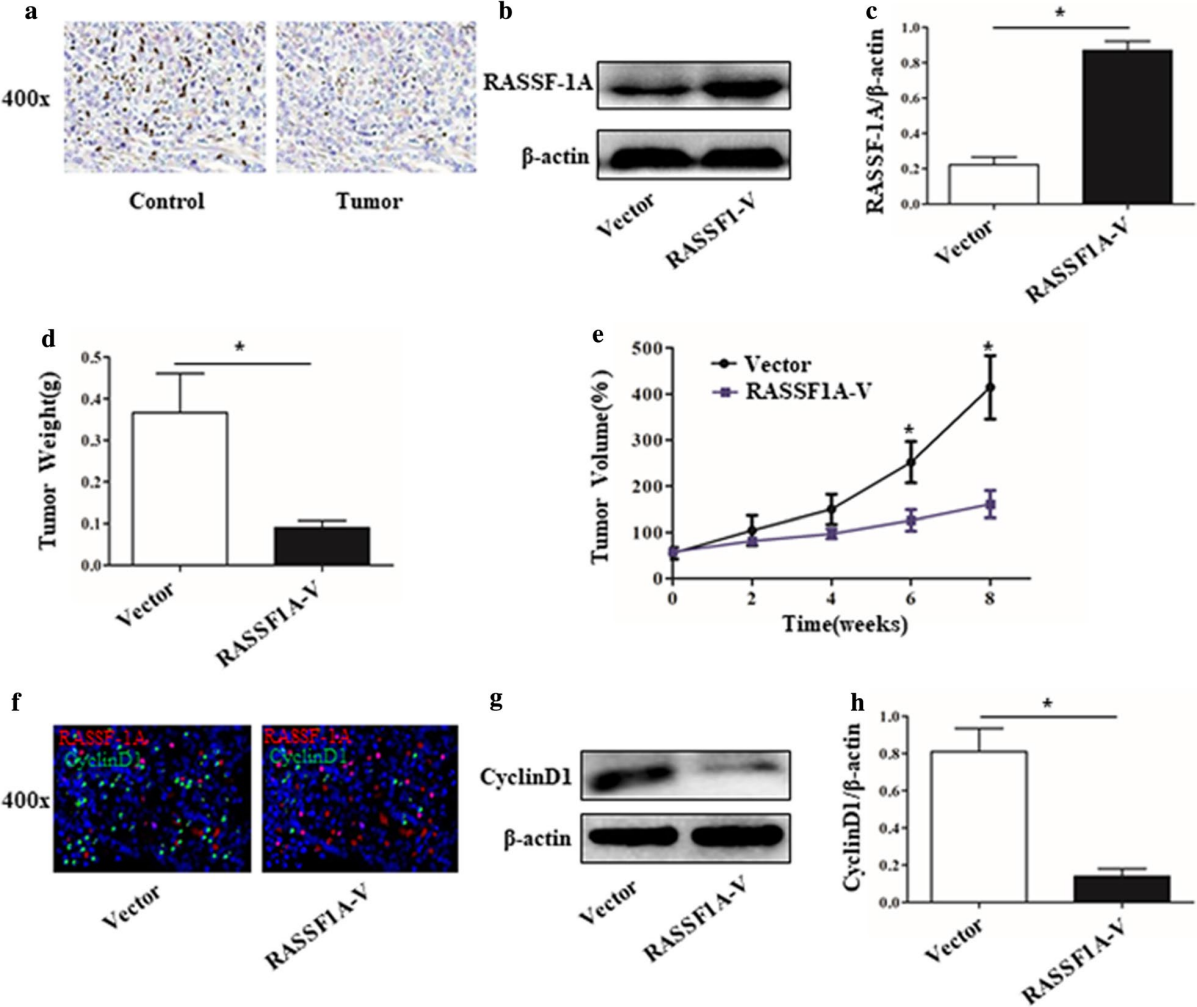
Overexpression of RASSF-1A gene in a mouse model of oral cancer inhibits tumor growth. **a** Immunohistochemical detection of RASSF-1A protein expression in normal mouse oral and oral cancer mouse tissues. **b**, **c** Western blot analysis of RASSF-1A gene expression in adenovirus overexpressing oral cancer mice (n = 6 in each group; *p < 0.05). **d** Mouse tumor tissue weight statistics after 8 weeks (n = 6 in each group; *p < 0.05). **e** Volume of mouse tumor tissue after 8 weeks (n = 6 in each group; *p < 0.05). **f** Immunofluorescence detection of CyclinD1 protein expression in mouse tumor tissues (RASSF-1A: red, CyclinD1: green); **g** Western blot analysis of CyclinD1 protein expression in mouse tumor tissues. **h** Statistical analysis of CyclinD1 protein expression in mouse tumor tissues detected by western blot (n = 6 in each group; *p < 0.05)

**Fig. 7:**
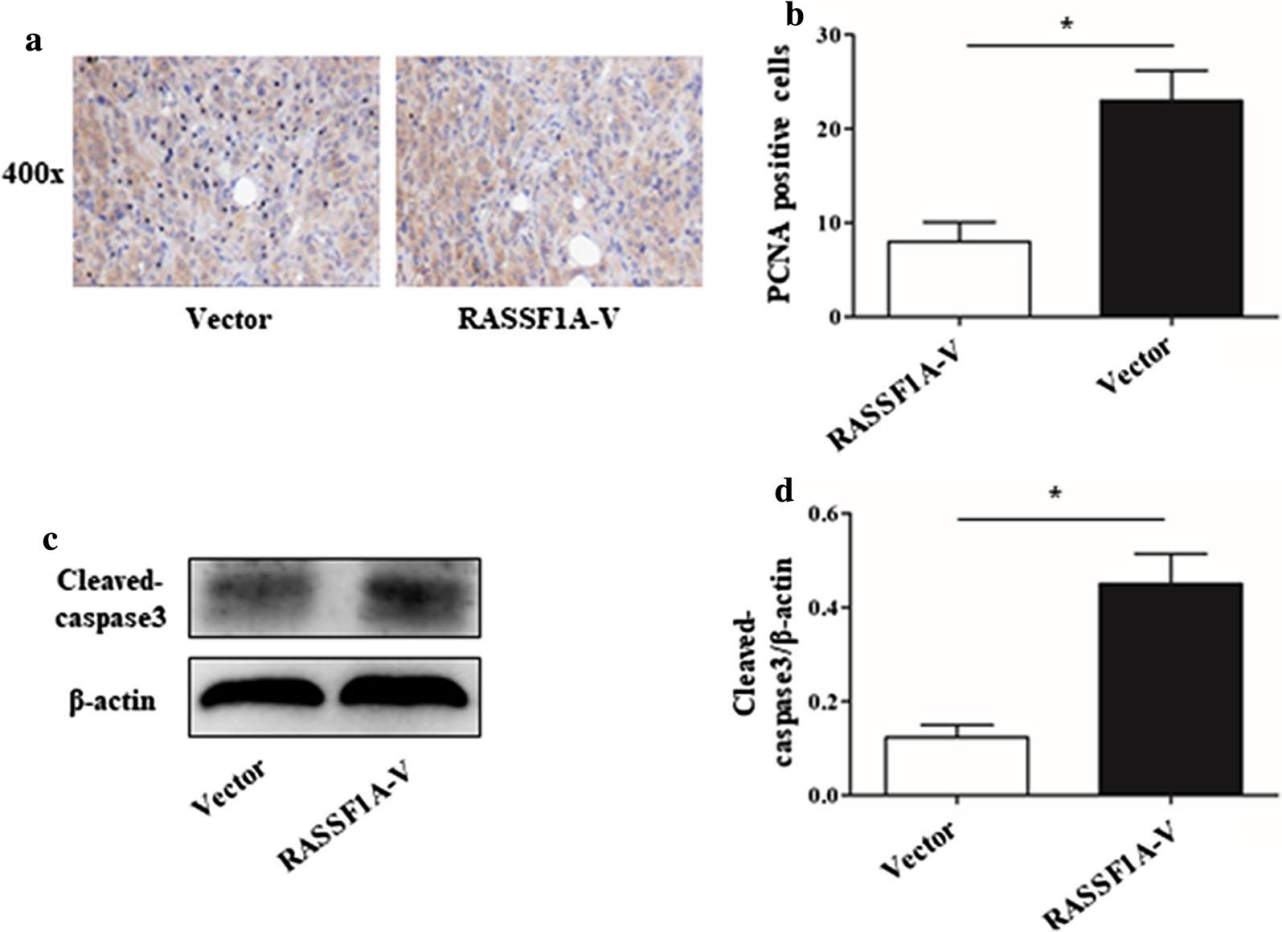
Overexpression of RASSF1A in oral squamous cell carcinoma of mice inhibits PCNA expression and promotes tumor cell apoptosis. **a** Immunohistochemical detection of PCNA protein expression in mice oral squamous cell carcinoma. ×400: image zoomed 400 times. **b** Immunohistochemical detection of PCNA protein positive cells in overexpressed mice oral squamous cell carcinoma (n = 6 in each group; *p < 0.05). **c** Western blot analysis of cleaved-caspase3 protein expression in mice oral squamous cell carcinoma. **d** Statistical analysis of the expression of caspase3 protein in mice oral squamous cell carcinoma by western blot (n = 6 in each group; *p < 0.05)

**Fig. 8:**
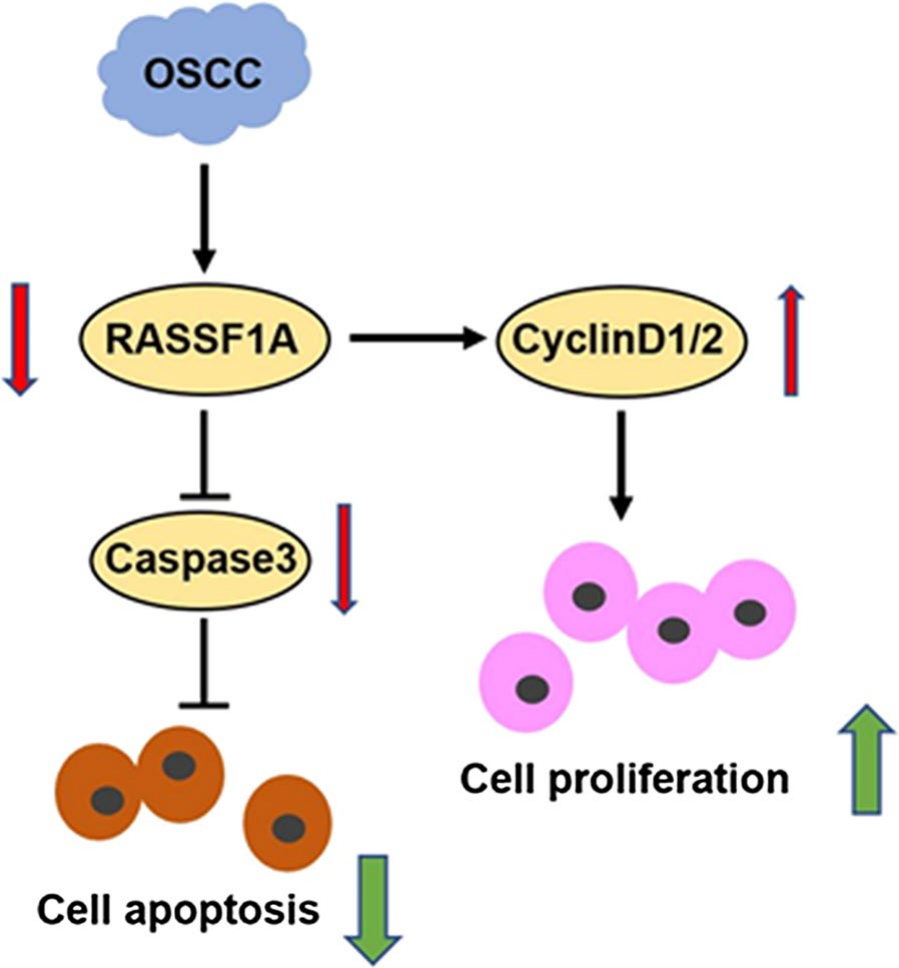
Schematic diagram of proposed mechanism

A mouse tongue squamous cell carcinoma model was established. The results of immunohistochemistry showed that RASSF-1A protein was highly expressed in tumor tissues (Fig. 6a). The RASSF-1A gene was overexpressed in the mouse by adenovirus, and the expression of RASSF-1A protein in the tumor tissue of the virus group was found to be increased by western blot and the difference was statistically significant (Fig. 6b, c). Control group mice infected with empty adenovirus. After 8 weeks of tumor formation, the weight and volume of the tumor tissues of the two groups of mice were taken out. The weight and volume of the tumor tissue overexpressing the RASSF-1A gene were significantly lower than those of the control group, with a significant difference (Fig. 6d, e). The immunofluorescence staining of tumor tissues of the two groups of mice showed that the expression of CyclinD1 (green) was increased in the tumor tissues of mice overexpressing RASSF-1A gene (Fig. 6f). At the same time, western blot analysis showed that the expression of CyclinD1 protein in the tumor tissues of the overexpressed mice increased, with a significant difference (Fig. 6g, h). The expression of proliferating protein PCNA was reduced in the tumor tissues of mice overexpressing RASSF1A gene (Fig. 7a, b). The expression of caspase3 active fragment was increased in tumor tissues of mice overexpressing RASSF1A gene (Fig. 7c, d). The mechanism of action was drawn as a schematic diagram. See Fig. 8.

## Discussion

Oral squamous cell carcinoma (OSCC), a malignant tumor of head and neck squamous cell carcinoma, has a high incidence and high mortality [16]. Current research shows that the development of OSCC is a multi-step evolution process involves a series of epigenetic changes. Head and neck squamous cell carcinoma is a major threat to global health problems [17]. It ranks 6th in common malignant tumors of the whole body and is one of the most common malignant tumors. Among them, oral cancer is common in all head and neck squamous cell carcinoma. OSCC is the most common type of oral tumors, accounting for more than 90% of oral malignant tumors, and 38% of head and neck cancer [17, 18]. The age of onset of OSCC is generally between 50 and 70 years old, mainly between 51 and 55 years old. In a few countries, the age of onset is mainly focused on 64 years old or older. Current treatments for OSCC include surgery, radiation therapy, and chemotherapy [19]. Although the corresponding diagnostic and clinical treatment strategies to prevent and treat OSCC, due to its high invasive ability, high metastasis rate and high recurrence rate, the survival rate of OSCC patients has not improved significantly, and the 5-year survival rate is still less than 50% [19, 20]. Molecular epidemiological studies have shown that OSCC is a multi-gene multi-step malignant tumor involving the activation of proto-oncogenes and the inactivation of tumor suppressor genes [21]. The current research results showed that hundreds of genes are involved in the development of OSCC, and new genes are constantly being studied, such as microRNA, P53, RARB2, and hnRNPK [22].

RASSF-1A has been reported to be overexpressed in malignant tumors of other organs of the human body, and CyclinD1 is one of the common tumor proliferation-related proteins reported by scholars at home and abroad. Two-factor association has been reported in prostate cancer tissues [13]. In OSCC tissues, the correlation between the two has not been reported. Current studies have demonstrated that cell cycle-dependent protein kinase 2A gene and RAS association domain family 1A (RASSF1A gene) promoter methylation play an important role in the early diagnosis and prognosis of non-small cell lung cancer [11, 23]. The RASSF1A gene is a tumor suppressor gene, and its methylation impairs the cell regulation mechanism and causes cancer through multiple downstream transcription pathways [24]. At present, RASSF1A is a novel tumor suppressor gene, and its specific mechanism of action in oral cancer is still unclear. ASSF1A is a member of the Ras effector family and can activate multiple signaling pathways in combination with different effectors [25, 26]. The Ras/RASSF1/ERK pathway transmits signals from outside the cell to the cell, mediating cell differentiation, proliferation, etc. The RASSF1A protein phosphorylates the kinase site of the ATM protein, thereby exerting a tumor suppressing effect. The study found that RASSF1A is methylated in esophageal cancer, gastric cancer, and prostate cancer, and the degree of methylation is related to clinical stage [27, 28]. It has been reported that the expression of RASSF1A in esophageal cancer tissues is associated with TNM stage, depth of invasion, and lymph node metastasis [23]. It plays an important role in the occurrence and development of esophageal cancer, which is consistent with the results of this group. It is suggested that RASSF1A is lowly expressed in esophageal squamous cell carcinoma tissues, which can accelerate the deterioration of cancer cells and promote the metastasis and invasion of cancer cells [29]. Kaplan–Meier single factor analysis showed that RASSF1A positive patients had higher survival time than negative patient. Therefore, abnormal expression of RASSF1A is associated with tumor progression, metastasis, and prognosis in patients, suggesting that RASSF1A expression is reduced, which can promote cancer cell deterioration and enhance cancer cell metastasis and invasion [30–32]. In our study, the low expression of RASSF-1A gene in OSCC was first discovered. In vitro, knockdown of RASSF-1A gene in tongue squamous cell carcinoma cells promoted cell proliferation and migration, inhibited apoptosis of cancer cells. It is suggested that RASSF-1A gene may be involved in the metastasis and proliferation of OSCC. At the same time, we detected tumor-associated proliferating proteins and found that high expression of CyclinD1 protein was observed in tumor cells that knocked down RASSF-1A. In vivo experiments also found that after overexpression of RASSF-1A gene in oral cancer model mice, tumor growth was slowed, tumor volume was decreased, and CyclinD1 protein expression in tumor tissues was decreased.

## Conclusions

Taken together, this study found that RASSF-1A gene is low expressed in OSCC, resulting in increased expression of CyclinD1 protein to promote tumor growth and metastasis. Therefore, it provides new ideas and directions for the treatment and prevention of OSCC.

## Data Availability

All data generated or analysed during this study are included in this published article.

## Abbreviations

OSCC: oral squamous cell carcinoma
FBS: fetal bovine serum
PI: propidium iodide
